# Double Negative Differential Resistance Device Based on Hafnium Disulfide/Pentacene Hybrid Structure

**DOI:** 10.1002/advs.202000991

**Published:** 2020-08-05

**Authors:** Kil‐Su Jung, Keun Heo, Min‐Je Kim, Maksim Andreev, Seunghwan Seo, Jin‐Ok Kim, Ji‐Hye Lim, Kwan‐Ho Kim, Sungho Kim, Ki Seok Kim, Geun Yong Yeom, Jeong Ho Cho, Jin‐Hong Park

**Affiliations:** ^1^ Department of Semiconductor and Display Engineering Sungkyunkwan University Suwon 440‐746 South Korea; ^2^ Memory Technology Design Team Samsung Electronics Co. Hwasung 18448 South Korea; ^3^ Department of Electrical and Computer Engineering Sungkyunkwan University Suwon 440‐746 South Korea; ^4^ SKKU Advanced Institute of Nano Technology (SAINT) Sungkyunkwan University Suwon 440‐746 South Korea; ^5^ Jet Propulsion Laboratory (JPL) California Institute of Technology Pasadena CA 91109 USA; ^6^ Research Laboratory of Electronics Massachusetts Institute of Technology (MIT) Cambridge MA 02139‐4307 USA; ^7^ School of Advanced Materials Science and Engineering Sungkyunkwan University Suwon 440‐746 South Korea; ^8^ Department of Chemical and Biomolecular Engineering Yonsei University Seoul 120‐749 South Korea

**Keywords:** HfS_2_, hybrid structures, negative differential resistance (NDR), pentacene

## Abstract

Recently, combinations of 2D van der Waals (2D vdW) materials and organic materials have attracted attention because they facilitate the formation of various heterojunctions with excellent interface quality owing to the absence of dangling bonds on their surface. In this work, a double negative differential resistance (D‐NDR) characteristic of a hybrid 2D vdW/organic tunneling device consisting of a hafnium disulfide/pentacene heterojunction and a 3D pentacene resistor is reported. This D‐NDR phenomenon is achieved by precisely controlling an NDR peak voltage with the pentacene resistor and then integrating two distinct NDR devices in parallel. Then, the operation of a controllable‐gain amplifier configured with the D‐NDR device and an n‐channel transistor is demonstrated using the Cadence Spectre simulation platform. The proposed D‐NDR device technology based on a hybrid 2D vdW/organic heterostructure provides a scientific foundation for various circuit applications that require the NDR phenomenon.

## Graphical abstract

In this work, a double negative differential resistance (D‐NDR) characteristic of a hybrid 2D van der Waals (2D vdW)/organic tunneling device consisting of a hafnium disulfide/pentacene heterojunction and a 3D pentacene resistor is reported. Then the operation of a controllable‐gain amplifier configured with the D‐NDR device and an n‐channel transistor is demonstrated using the Cadence Spectre simulation platform.

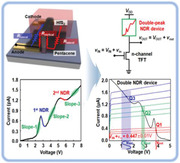

## 1 Introduction

Various types of heterojunctions such as type‐I (straddling gap), type‐II (staggered gap), and type‐III (broken gap), which differ with regard to their energy‐band alignment, have been exploited to implement diverse functionalities in modern electronic and optoelectronic devices. For instance, type‐I heterojunctions were used to implement a quantum‐well structure to confine carriers for efficient light‐emitting diode^[^ ^1^, ^2^, ^3^ ^]^ and laser diode devices.^[^ ^4^ ^]^ Additionally, a resonant tunneling diode, featuring the negative differential resistance (NDR) phenomenon, was implemented via a quantum‐well structure comprising type‐I heterojunctions (dielectric/semiconductor).^[^ ^5^, ^6^, ^7^, ^8^ ^]^ A type‐II heterojunction structure confined electrons well in a varied channel region, thereby facilitating the implementation of a high‐electron‐mobility transistor.^[^ ^9^, ^10^ ^]^ An Esaki tunneling diode, presenting the NDR phenomenon, was fabricated on the basis of type‐III heterojunctions.^[^ ^11^, ^12^, ^13^, ^14^, ^15^ ^]^ However, it is very difficult to fabricate the aforementioned heterojunctions with excellent interface quality by using epitaxial growth techniques of conventional semiconductor and dielectric materials. This is because the lattice mismatch between the materials causes numerous crystal defects that function as traps, thereby leading to generation, recombination, and tunneling currents in the heterojunction regions. Thus, 2D van der Waals (2D vdW) materials, such as molybdenum disulfide (MoS_2_), tungsten diselenide (WSe_2_), black phosphorus, and hexagonal boron nitride (*h*‐BN), have been proposed for devices that require heterojunction structures. Their defect‐free surface, with the absence of dangling bonds, facilitates the formation of a sharp and clean interfacial heterojunction.^[^ ^16^, ^17^, ^18^, ^19^, ^20^ ^]^ Although various 2D vdW materials have been synthesized on the wafer scale via chemical vapor deposition,^[^ ^21^, ^22^, ^23^, ^24^, ^25^, ^26^, ^27^ ^]^ pulsed laser deposition,^[^ ^28^, ^29^, ^30^, ^31^ ^]^ and magnetron sputtering,^[^ ^32^, ^33^, ^34^, ^35^ ^]^ the complex heterojunction structures have not been fabricated with such 2D vdW crystal films. Therefore, in consideration of the current growth technology for 2D vdW materials, the adoption of organic materials without surface defects is a very attractive option. Because these organic materials are also free of surface defects and native surface‐oxidation issues, it is possible to achieve various high‐quality heterojunctions over a large area by stacking organic layers on 2D vdW crystal layers. For example, a hybrid solar cell with a MoS_2_/pentacene heterojunction exhibited an excellent photovoltaic effect,^[^ ^36^, ^37^, ^38^ ^]^ and an organic transistor fabricated by epitaxially growing a rubene channel film on an *h*‐BN dielectric layer exhibited a very high carrier mobility.^[^ ^39^, ^40^ ^]^

In this paper, we report a double‐NDR device fabricated on a 3D hybrid structure consisting of two 2D vdW/organic heterojunctions and one organic resistor. We used hafnium disulfide (HfS_2_) and pentacene as the 2D vdW and organic materials, respectively. The double‐NDR phenomenon was achieved via the following strategy: i) intentionally shifting the NDR peak position using a vertical organic resistor, and then, ii) integrating two NDR devices in parallel. By performing an analog circuit simulation, we verified the applicability of this double‐NDR device to a controllable‐gain amplifier circuit that was configured with the double‐NDR device and one n‐channel transistor. Additionally, the crystallinity of the pentacene film deposited on HfS_2_ and the alignment of the HfS_2_/pentacene heterojunction were investigated in detail via Raman analysis, atomic force microscopy (AFM), and X‐ray photoelectron spectroscopy (XPS).

## 2 Results and Discussion

**Figure 1:**
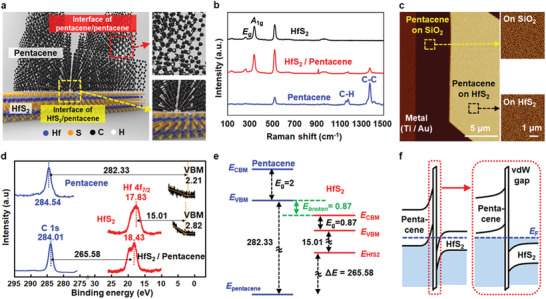
a) Schematic of the hybrid HfS_2_/pentacene heterojunction, showing the interfaces of HfS_2_/pentacene and pentacene/pentacene. b) Raman spectra of the HfS_2_, pentacene, and overlapped HfS_2_/pentacene regions. c) AFM image of a pentacene layer on SiO_2_ and HfS_2_. d) Core‐level X‐ray photoelectron spectra of the individual materials (HfS_2_, pentacene) and heterojunctions (HfS_2_/pentacene). Energy‐band alignments of HfS_2_ and pentacene in the equilibrium state e) before and f) after the formation of the contact. The information on the energy bands was obtained from the previous XPS analysis.

**Figure** **1**a shows a schematic of the HfS_2_/pentacene heterojunction, which is the core part of the 3D hybrid structure. A thin HfS_2_ layer was transferred onto a SiO_2_/Si substrate via a mechanical exfoliation method. Then, an active junction area was defined on the HfS_2_ via electron‐beam (e‐beam) lithography, followed by the deposition of a pentacene layer using a thermal evaporator (details are presented in Section S1 of the Supporting Information). Although a polycrystalline pentacene layer was predicted to be formed on the HfS_2_, similar to the case of depositing pentacene on SiO_2_, the interface between pentacene and HfS_2_ was free of crystal defects, which cause recombination/generation currents. This is because the surface of the HfS_2_ and the crystal grain of the pentacene did not have unterminated bonds, which facilitated the formation of a HfS_2_/pentacene junction with an atomically sharp interface. To confirm the formation of the HfS_2_/pentacene heterojunction, Raman analysis was conducted on three distinct regions of the sample: HfS_2_, pentacene, and the overlapped regions (Figure 1b). Raman peaks related to the HfS_2_ were observed at 263 and 338 cm^−1^, corresponding to the in‐plane vibration of Hf and S atoms (E_g_) and the out‐of‐plane vibration of S atoms (A_1g_), respectively.^[^ ^41^, ^42^ ^]^ For the pentacene region, three prominent peaks were observed. Two peaks at 1157 and 1178 cm^−1^ originated from the C—H in‐plane molecular vibration of the pentacene molecules, and the third peak at 1374 cm^−1^ was due to the asymmetric C—C stretching vibration along the short molecular axis in the molecular plane.^[^ ^43^, ^44^ ^]^ These five peaks also appeared for the overlapped region, and no shift or degradation was observed, thereby indicating the successful formation of the HfS_2_/pentacene heterojunction via van der Waals bonding. Furthermore, we verified the clean interface of the HfS_2_/pentacene heterojunction through cross‐sectional transmission electron microscopy (X‐TEM) and energy‐dispersive X‐ray spectroscopy measurements (see Figure S2 in the Supporting Information). As shown in Figure 1c, AFM analysis confirmed that the pentacene deposited on the HfS_2_ and the SiO_2_ exhibited similar grain sizes (247 nm on HfS_2_ and 244 nm on SiO_2_) and surface‐roughness values (5.78 on HfS_2_ and 5.56 nm on SiO_2_). The thicknesses of the HfS_2_ and pentacene layers were ≈37.5 nm and ≈50 nm, respectively. We then performed XPS measurements to predict precisely the energy band alignment of the HfS_2_/pentacene heterojunction. Figure 1d presents i) core‐level (CL) spectra of Hf 4f and C 1s; ii) CL spectra of Hf 4f + C 1s; and iii) valence band maximum (VBM) for HfS_2_, pentacene, and HfS_2_/pentacene heterojunction. Based on the CL spectra analysis of the individual materials and the heterojunction, we estimated the valence band offset (VBO) of the HfS_2_/pentacene heterojunction using the following equation: VBO = (*E*_Pentacene_ − *E*_VBM_Pentacene_) − (*E*_HfS2_ − *E*_VBM_HfS2_) − Δ*E*, where *E*_Pentacene_ and *E*_HfS2_ represent the CL binding energies of pentacene and HfS_2_, respectively. *E*_VBM_Pentacene_, *E*_VBM_HfS2_, and Δ*E* represent the VBM of pentacene, the VBM of HfS_2_, and the binding energy difference between HfS_2_ and pentacene, respectively.^[^ ^45^, ^46^, ^47^ ^]^ The VBO of HfS_2_/pentacene heterojunction was calculated to be 1.74 eV. We then found the broken‐gap energy of 0.87 eV by subtracting the HfS_2_ bandgap (0.87 eV) from the obtained VBO value (1.74 eV). Using the VBM, the broken‐gap energy, and the known energy band information for HfS_2_^[^ ^48^, ^49^ ^]^ and pentacene,^[^ ^50^, ^51^, ^52^ ^]^ we completed the energy band alignment of HfS_2_/pentacene heterojunction, as shown in Figure 1e. Figure 1f shows the energy band structures of the HfS_2_/pentacene heterojunction after the contact of the two materials. Because the highest occupied molecular orbital (HOMO) level of pentacene is above the conduction‐band minimum of HfS_2_ (type‐III heterojunction), large amounts of holes and electrons are expected to accumulate in the pentacene and HfS_2_ regions near the interface, respectively. This energy band property facilitates the formation of a highly doped n^+^/p^+^ heterojunction without the assistance of additional doping such as chemical doping^[^ ^53^ ^]^ or electrostatic doping.^[^ ^54^, ^55^, ^56^ ^]^ In addition to the HfS_2_, we also attempted to utilize ZrS_2_, SnS_2_, and SnSe_2_ to develop a type‐III heterojunction. The selected 2D vdW materials were expected to form a broken gap with pentacene because their conduction‐band minimum levels were lower than pentacene's HOMO level. However, for unknown reasons, no other junctions except the HfS_2_/pentacene junction presented the NDR phenomenon (for details see Figure S4 in the Supporting Information). Meanwhile, various efforts have been made to synthesize the HfS_2_ 2D vdW film since a HfS_2_ film was reported on a *c*‐plane sapphire substrate by chemical vapor deposition (CVD) using HfCl_4_ and S powders in 2017.^[^ ^57^, ^58^, ^59^ ^]^ If the HfS_2_ flake is replaced by the HfS_2_ CVD films via the synthesis techniques later, it would be possible to scale up the proposed double‐NDR device to circuit level.

**Figure 2:**
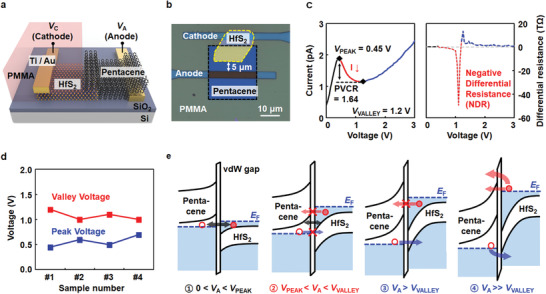
a) Schematic of the NDR device based on the hybrid HfS_2_/pentacene heterojunction. b) Optical image of the HfS_2_/pentacene hybrid NDR device. c) *I*–*V* characteristic curve of the hybrid NDR device on a linear scale (left) and the corresponding differential resistance curve (right). d) Peak (blue square mark) and valley (red square mark) voltage values of four different hybrid NDR devices. e) Energy‐band diagrams of the hybrid NDR device in different operating‐voltage regions.

To investigate the proposed HfS_2_/pentacene heterojunction in detail, we performed electrical measurements for the heterojunction device shown in **Figure** **2**a. The overlapped junction area was ≈122 µm^2^, and the anode was formed 5 µm away from the edge of the HfS_2_/pentacene heterojunction (see the optical image in Figure 2b). Figure 2c presents the current–voltage (*I*–*V*) characteristic curve on a linear scale (left) measured at room temperature (300 K) and the corresponding differential resistance values (right). The peak voltage (*V*_PEAK_) and valley voltage (*V*_VALLEY_) of the NDR curve were 0.45 and 1.2 V, respectively, and the peak‐to‐valley ratio (PVCR) was 1.64. The red line in the right figure represents the NDR values calculated from the NDR curve between *V*_PEAK_ and *V*_VALLEY_. The average values of *V*_PEAK_ and *V*_VALLEY_ that were measured for four different devices were 0.56 and 1.08 V, respectively, and the standard deviations were 0.11 and 0.10 in Figure 2d, respectively (details are presented in Section S5 of the Supporting Information). The operating mechanism of the NDR device was illustrated with energy band diagrams for different anode voltage regions, as shown in Figure 2e. At a low positive anode voltage (region 1; 0 < *V*_A_ < *V*_PEAK_ = 0.45 V), free electrons in the conduction band of the n‐type HfS_2_ tunnel into empty states in the valence band of the p‐type pentacene through the vdW gap,^[^ ^14^ ^]^ thereby increasing the current. This tunneling current continues to increase until the Fermi level of HfS_2_ aligns with the HOMO level of pentacene. If the anode voltage increases further (region 2; *V*_PEAK_ = 0.45 V < *V*_A_ < *V*_VALLEY_ = 1.2 V), the filled states in the conduction band of HfS_2_ are located into the bandgap of pentacene, and the transport of holes from pentacene to HfS_2_ is blocked, reducing the current. Thus, the tunneling current is maximized (≈1.88 pA) at 0.45 V. At a higher anode voltage of >*V*_VALLEY_ (region 3; *V*_A_ > *V*_VALLEY_ = 1.2 V), owing to the relatively small bandgap of HfS_2_ (0.87 eV), the holes start to move at first from the HOMO band of pentacene to the filled states in the valence band of HfS_2_ via thermionic emission or Fowler–Nordheim (FN) tunneling, thereby increasing the current again.^[^ ^15^, ^60^ ^]^ However, the electrons in the conduction band of HfS_2_ are still blocked by the bandgap of pentacene under this bias condition. At a higher bias, in region 4 (*V*_A_ ≫ *V*_VALLEY_ = 1.2 V), the thermionic emission or FN tunneling of electrons from the filled states in the HfS_2_ conduction band to the empty states above the lowest unoccupied molecular orbital (LUMO) of pentacene becomes dominant, thereby increasing the current continuously. We additionally investigated the temperature‐dependent electrical characteristics of the HfS_2_/pentacene NDR device, as provided in Figure S6 (Supporting Information).

**Figure 3:**
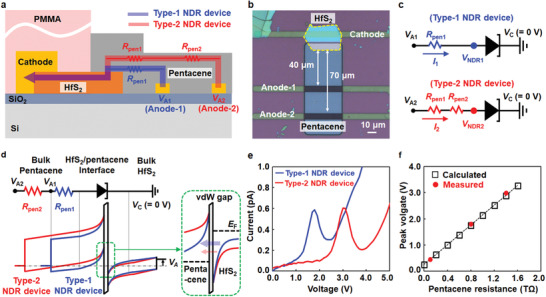
a) Cross‐sectional schematic of type‐1 and type‐2 NDR devices with different pentacene resistors and b) a corresponding optical image. c) Equivalent circuits, d) energy‐band diagrams, and e) *I*–*V* characteristic curves for the hybrid NDR devices. f) Calculated (black squares) and measured (red circles) peak voltages with respect to the pentacene resistance.

To increase the number of peaks for the NDR device, we controlled the peak and valley positions by introducing an additional lateral resistor in the NDR path. Because the sheet resistance of HfS_2_ (3 GΩ sq^−1^) is significantly smaller than that of pentacene (600 GΩ sq^−1^), the pentacene was selected for the control resistor for the NDR peak/valley shift. Detailed information regarding the resistance evaluation is presented in Section S7 (Supporting Information). **Figure** **3**a displays the current paths of two types of NDR devices, which present i) the first NDR current path through the pentacene resistance (*R*_pen1_, 0.8 TΩ) and ii) the second path including the additional lateral pentacene resistor (*R*_pen1_ + *R*_pen2_, 1.4 TΩ). An optical image showing the two current paths for the NDR device is presented in Figure 3b. The length, width, and thickness of the additional pentacene resistors (*R*_pen2_) are 30, 50, and 50 nm, respectively, resulting in a resistance value of 0.6 TΩ. The peak/valley shift mechanism can be explained by exploiting the two equivalent NDR circuits shown in Figure 3c. The voltage drops at the HfS_2_/pentacene heterojunction in the first and second NDR current paths can be determined using the equations *V*_NDR1_ = *V*_A1_ – (*R*_pen1_ · *I*_1_) and *V*_NDR2_ = *V*_A2_ – ((*R*_pen1_ + *R*_pen2_) · *I*_2_), respectively. Owing to the voltage‐division effect caused by the additional lateral pentacene resistance (*R*_pen2_), *V*_NDR2_ is generally lower than *V*_NDR1_. This reduction in *V*_NDR2_ decreases the energy band bending (red line) at the HfS_2_/pentacene heterojunction interface, which is a major cause of the NDR phenomenon (Figure 3d). The reduced energy band bending causes less electrons from the HfS_2_ to tunnel into the empty valence‐band states of pentacene, which reduces the tunneling current and eventually results in the formation of the second NDR peak (Figure 3e). Therefore, the insertion of the pentacene lateral resistor (0.6 TΩ) resulted in shifted peak/valley voltages (3.0/3.8 V), compared with the values of the first NDR curve (1.8/2.5 V). Figure 3f presents the peak voltage shifting results with respect to the pentacene resistance. The red circles and black squares represent the peak voltage shift values extracted from the experimentally measured data and the theoretically calculated data, respectively. The peak voltage shift increased gradually with an increase in the pentacene resistance. The two results were well fitted, indicating the precise controllability of the proposed peak shifting method.

**Figure 4:**
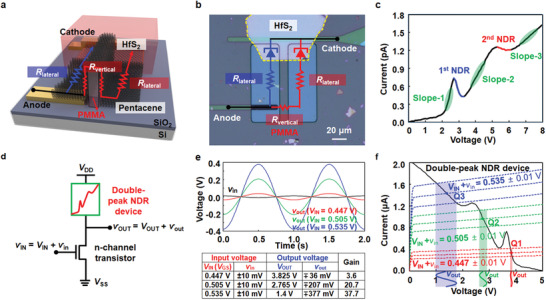
a) Schematic of the double‐peak hybrid NDR device consisting of two HfS_2_/pentacene heterojunctions and one 3D pentacene resistor. b) Optical image of the double‐NDR device, where the equivalent circuits are indicated. c) *I*–*V* characteristic curve of the double‐NDR device. d) Circuit configuration of a controllable‐gain amplifier integrated with the double‐NDR device and an n‐channel transistor. e) Output AC signal (*v*_out_, red/green/blue solid lines) corresponding to the input AC signal (*v*_IN_, black solid line) under three different DC *V*_IN_ conditions (0.447/0.505/0.535 V). Summary table showing the input (*v*_IN_ = *V*_IN_ + *v*_in_) and output (*v*_OUT_ = *V*_OUT_ + *v*_out_) voltage conditions and the corresponding gain values. f) Load‐line analysis of the controllable‐gain amplifier circuit presenting amplified output AC signals at three different operating points (*V*_IN_ ± *v*_in_: 0.447 ± 0.01, 0.505 ± 0.01, and 0.535 ± 0.01 V).

Using this peak shifting method, we demonstrated a double‐peak NDR device, as shown in **Figure** **4**a, which consisted of two HfS_2_/pentacene junctions with different current paths connected in parallel. The current through the first NDR path was dominated by the lateral pentacene resistance (*R*_lateral_), but that through the second NDR path was mainly influenced by the vertical pentacene resistance (*R*_vertical_), which was formed by depositing pentacene at the side walls of the Poly methyl methacrylate (PMMA) structure. The usage of pentacene resistors causes the shift of the current peaks, and integration with HfS_2_/pentacene junctions allows the formation of multiple current peaks in a single device. Figure 4b presents an optical image of the double‐peak NDR device, which includes two parallel HfS_2_/pentacene junction diodes and the pentacene resistors. Because the two junctions have similar junction areas, the length of the current path in the pentacene region is a dominant parameter inducing the peak shift. Figure 4c shows the measured double‐peak NDR characteristic curves. The first NDR peak/valley voltages determined by *R*_lateral_ were 2.7/3.2 V (PVCR: 2.2), and the values of the second NDR determined by *R*_lateral_ + *R*_vertical_ were 5.3/6.1 V (PVCR: 1.4). The second peak current was larger than the first one because the diffusion current of the first junction appeared. The *I*–*V* characteristic curve exhibited three distinct slopes (d*I*/d*V*) according to the carrier‐transport mechanism. First, slope‐1 is dominated by the tunneling currents flowing through the first and second current paths. Second, the current in the slope‐2 region consists of the diffusion current of the first path and the tunneling current of the second path. Lastly, slope‐3 can be explained by the sum of the diffusion currents through the first and second paths. Because the slope corresponding to the diffusion current is smaller than that corresponding to the tunneling current,^[^ ^15^ ^]^ the current slope decreases as the applied voltage increases (details are presented in Section S8 of the Supporting Information). Consequently, the double‐peak NDR device not only had two NDR regions but also had three different slopes with positive differential resistances. Additionally, we investigated the NDR characteristics with respect to energy bandgap and electron affinity of HfS_2_, as shown in Figure S9 (Supporting Information).

Finally, we implemented a controllable‐gain amplifier using the multiple *I*–*V* slopes, as shown in Figure 4d. In this amplifier circuit, we achieved three different gains by controlling *V*_GS_. This was done by integrating an HfS_2_/pentacene heterojunction NDR device as a load resistor with an n‐channel transistor as a driver, where the total resistance in the n‐channel transistor could be controlled by changing the applied gate voltage. The n‐channel transistor was obtained from a library of the simulation tool Cadence (the details are reported in Section S10 of the Supporting Information), and the current level of the double‐peak NDR was intentionally increased for the proof‐of‐concept of a controllable‐gain amplifier circuit. The low current level of the double NDR device can be further increased if the heterojunctions are constructed with films having a similar energy band structure and higher conductance. As a proof, the peak current of the HfS_2_/BP heterojunction device was 3.7 nA, which was 2000 times higher than that of the HfS_2_/pentacene heterojunction. The supply voltage (*V*_DD_ = 5 V) was connected to the cathode of the double‐peak NDR device, and the source of the n‐channel transistor was grounded (*V*_SS_ = 0 V). The input voltage (*v*_IN_ = *V*_IN_ + *v*_in_) was applied to the gate of the n‐channel transistor, and then we measured the output voltage (*v*_OUT_ = *V*_OUT_ + *v*_out_) at the node shared by the two devices. Figure 4e shows the small output signals for input sinusoid signals with a magnitude of 10 mV in the controllable‐gain amplifier, which were examined for three different direct‐current (DC) *V*_IN_ (= *V*_GS_) conditions. By selecting these three *V*_IN_ values, we made the driver current of the n‐channel transistor have an operating point on three different resistance lines of the double‐peak NDR device. When the *V*_IN_ values were 0.447, 0.505, and 0.535 V, the *v*_out_ signal corresponding to the *v*_in_ signal with a magnitude of ±10 mV fluctuated with peak‐to‐peak values of i) ∓36 mV, ii) ∓207 mV, and iii) ∓377 mV, respectively. Consequently, we confirmed three different alternating‐current (AC) voltage‐gain values: 3.6, 20.7, and 37.7 V/V for the *V*_IN_ values of 0.447, 0.505, and 0.535 V, respectively. Figure 4f shows a load‐line analysis explaining the operation mechanism of the controllable‐gain amplifier circuit. The *I*–*V* characteristics of the double‐peak NDR device and n‐channel transistor are represented by solid and dashed lines, respectively. To make the driving‐current curve of the n‐channel transistor intersect the three *I*–*V* sloped lines of the double‐peak NDR load, we applied three different *V*_IN_ values: 0.447, 0.505, and 0.535 V. When a low *V*_IN_ was applied to 0.447 V, the driving current intersected with the steepest slope‐1 region of the double‐NDR device. Consequently, the first operating point (Q1) was stabilized at the point with a current of 0.337 µA and a voltage of 3.825 V. At this Q1 point, the smallest *v*_out_ signal (∓36 mV, red solid line) corresponding to the *v*_in_ signal (±10 mV) was obtained owing to the largest *I*–*V* slope of the double‐NDR device. When an intermediate *V*_IN_ of 0.505 V was applied to obtain an operating point in the slope‐2 region, the current flowing through the double‐NDR device and the corresponding voltage were 0.962 µA and 2.765 V, respectively (Q2, the second operating point). At point Q2, the *v*_out_ signal for a *v*_in_ signal with a magnitude of ±10 mV was amplified to ∓207 mV (green solid line). Finally, when a high *V*_IN_ of 0.535 V was applied to the n‐channel transistor, the current and voltage of the third operating point (Q3) were 1.432 µA and 1.4 V, respectively, and Q3 appeared in the *I*–*V* slope‐3 region. Here, we observed the largest fluctuation of the *v*_out_ signal (∓377 mV, blue solid line) for the same *v*_in_ signal. A detailed explanation about the operation of the controllable‐gain amplifier is also provided in Figure S11 (Supporting Information). Furthermore, we confirmed that the proposed controllable‐gain amplifier could operate even for the 10 MHz input signal (Figure S12, Supporting Information).

## 3 Conclusions

We implemented the NDR phenomenon in a hybrid 2D vdW/organic tunneling device consisting of a HfS_2_/pentacene heterojunction structure. This phenomenon was achieved by forming a broken‐gap band structure without the assistance of additional doping such as chemical or electrostatic doping. The broken‐gap band alignment of the HfS_2_/pentacene heterojunction was experimentally examined through XPS‐based CL spectra analysis of the 2D vdW and organic materials. Additionally, we controlled the peak and valley positions in the NDR characteristic curves by introducing an additional lateral resistor, thereby resulting in peak/valley shifts of 1.2/1.3 V. Finally, we demonstrated a double‐NDR device with hybrid structures consisting of two HfS_2_/pentacene heterojunctions and one 3D pentacene resistor. The double‐NDR phenomenon was achieved by shifting the NDR peak voltage with the 3D pentacene resistor and then integrating two NDR devices in parallel. Thus, we obtained a double‐peak NDR device with first NDR peak/valley voltages of 2.7/3.2 V and second NDR peak/valley voltages of 5.3/6.1 V. This double‐NDR device featured not only two NDR regions but also three different regions with positive differential resistances. Using the three positive *I*–*V* slope regions, we theoretically implemented a controllable‐gain amplifier circuit consisting of one double‐NDR device and one n‐channel transistor. The amplified *v*_out_ signal corresponding to the *v*_in_ signal (±10 mV) exhibited peak‐to‐peak values of ∓36, ∓207, and ∓377 mV, and voltage‐gain values of 3.6, 20.7, and 37.7 V/V for three *V*_IN_ values of 0.447, 0.505, and 0.535 V, respectively.

## 4 Experimental Section

##### Fabrication of Hybrid HfS_2_/Pentacene Heterojunction‐Based NDR Device

An HfS_2_ flake was mechanically exfoliated onto a 90 nm thick SiO_2_/Si substrate. Subsequently, an anode and a cathode were patterned via a photolithography process, and Ti/Au (10/30 nm) layers were deposited using an e‐beam evaporation system, followed by a liftoff process. An e‐beam lithography process was then applied to define the HfS_2_/pentacene heterojunction area. Finally, a pentacene film was deposited using a thermal evaporation system.

##### Characterization of HfS_2_/Pentacene Heterojunctions

Raman spectroscopy analysis was performed in the HfS_2_, pentacene, and HfS_2_/pentacene overlapped regions using a WITec micro‐Raman spectrometer system with a frequency‐doubled neodymium‐doped yttrium aluminum garnet (Nd–YAG) laser beam (532 nm laser excitation). Additionally, AFM was conducted in the HfS_2_/pentacene heterojunction region using an NX10 (Park Systems Corp.). To investigate the core levels of the HfS_2_ and pentacene materials, XPS information was extracted using a MultiLab 2000 (Thermo VG, Mg K*α* source) after the peak energies were calibrated with respect to the C 1s peak at 284.5 eV. A depth profiling analysis was conducted in the small‐area XPS mode, where a 3 mm × 3 mm area was etched for 30 s using an in situ Ar^+^‐ion gun. The electrical properties of the HfS_2_/pentacene NDR device were investigated under vacuum conditions at room temperature by using a semiconductor parameter analyzer (Keithley 4200) connected to a probe station.

##### Simulation of Controllable‐Gain Amplifier

By using the electrical characteristics of the double‐NDR device, a lookup table‐based model of the double‐NDR device was developed in the Verilog‐A language for a circuit simulation. Then the DC and AC operations of the controllable‐gain amplifier circuit consisting of the double‐NDR device and the n‐channel transistor was investigated.

## Conflict of Interest

The authors declare no conflict of interest.

## Supporting information

Supporting Information
