# Supplementary material for: Double Negative Differential Resistance Device Based on Hafnium Disulfide/Pentacene Hybrid Structure
Source: Adv Sci (Weinh). 2020 Aug 5;7(19):2000991. doi: 10.1002/advs.202000991 (PMC7539188; doi:10.1002/advs.202000991)
Supplement: Supplementary file 1 — Supporting Information [file ADVS-7-2000991-s001.pdf]

© 2020 Wiley-VCH GmbH

ADVANCED SCIENCE
Open Access

Supporting Information

for *Adv. Sci.,* DOI: 10.1002/advs.202000991

Double Negative Differential Resistance Device based on Hafnium Disulfide / Pentacene Hybrid Structure

Kil-Su Jung, Keun Heo, Min-Je Kim, Maksim Andreev, Seunghwan Seo, Jin-Ok Kim, Ji-Hye Lim, Kwan-Ho Kim, Sungho Kim, Ki Seok Kim, Geun Yong Yeom, Jeong Ho Cho, and Jin-Hong Park**

DOI: 10.1002/((please add manuscript number))
Article type: Communication

Supporting Information

Double Negative Differential Resistance Device based on Hafnium Disulfide / Pentacene Hybrid Structure

Kil-Su Jung, Keun Heo, Min-Je Kim, Maksim Andreev, Seunghwan Seo, Jin-Ok Kim, Ji-Hye Lim, Kwan-Ho Kim, Sungho Kim, Ki Seok Kim, Geun Yong Yeom, Jeong Ho Cho, and Jin-Hong Park**

K.-S. Jung, Prof. J.-H. Park
Department of Semiconductor and Display Engineering, Sungkyunkwan University, Suwon 440-746, South Korea
E-mail: jhpark9@skku.edu

K.-S. Jung
Memory Technology Design Team, Samsung Electronics Co., Hwasung 18448, South Korea

K. Heo, M. Andreev, S. Seo, J.-O. Kim, J.-H. Lim, K.-H. Kim, Prof. J.-H. Park
School of Electronic and Electrical Engineering, Sungkyunkwan University, Suwon 440-746, South Korea

M.-J. Kim
SKKU Advanced Institute of Nano Technology (SAINT), Sungkyunkwan University, Suwon 440-746, South Korea

S. Kim
Jet Propulsion Laboratory (JPL), California Institute of Technology, Pasadena

K. S. Kim
Research Laboratory of Electronics, Massachusetts Institute of Technology (MIT), Cambridge, MA, USA

K.S. Kim, Prof. G. Y. Yeom
School of Advanced Materials Science and Engineering, Suwon 440-746, South Korea

Prof. J. H. Cho
Department of Chemical and Biomolecular Engineering, Yonsei University, Seoul 120-749, South Korea
E-mail: jhcho94@yonsei.ac.kr

Keywords: negative differential resistance (NDR), pentacene, HfS_2, hybrid structure

Fabrication process of HfS_2/pentacene heterojunction structure

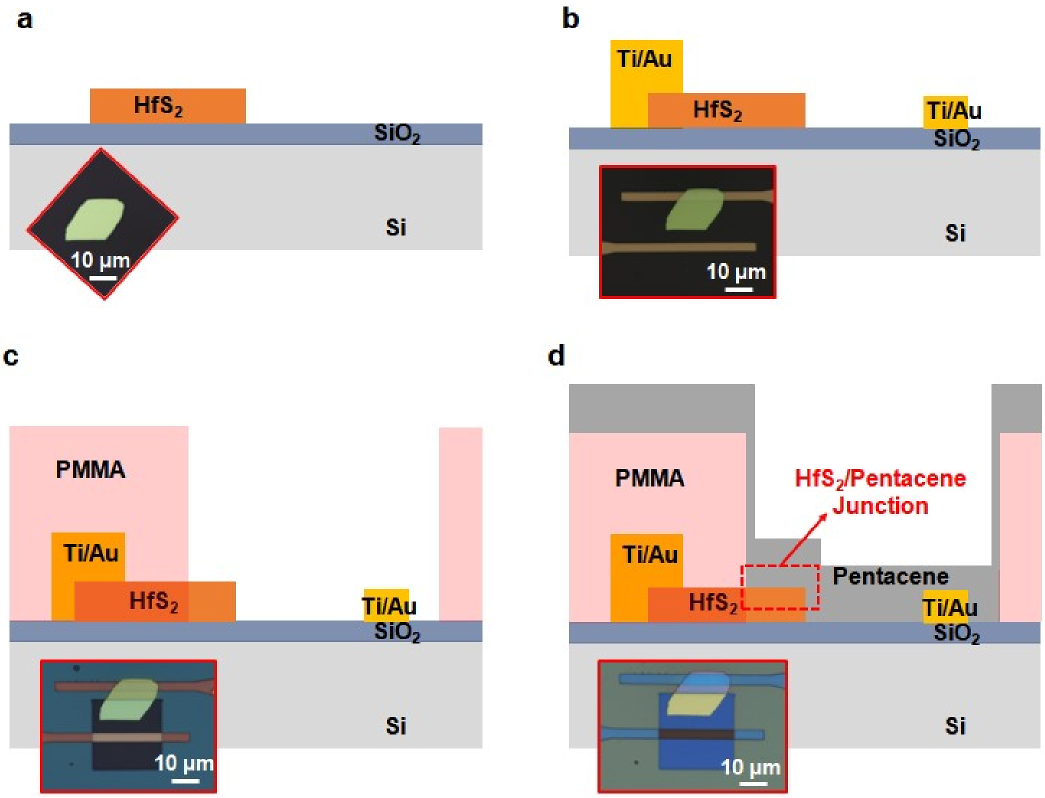

Figure S1. (a) Exfoliation of HfS_2 flake onto SiO_2/Si substrate by using adhesive tape. (b) Formation of Ti/Au (10/30 nm) electrode by photolithography and lift-off process. (c) HfS_2/pentacene heterojunction patterned by e-beam lithography process. (d) Deposition of pentacene film using a thermal evaporator.

Figures S1 (a) - (d) illustrate the fabrication process of HfS_2/pentacene heterojunction structure with insets of optical images for each step. The HfS_2 flake was mechanically exfoliated onto a 90nm thick SiO_2/Si substrate by using an adhesive tape in Figure S1 (a). The electrodes were patterned by photolithography, and Ti/Au (10/30nm) layers were deposited using an e-beam evaporation method. Then, a lift-off process was conducted in an acetone bath for 1h. The formed metal electrodes are shown in Figure S1 (b). Next, the HfS_2/pentacene heterojunction regions were defined by PMMA pattern via an e-beam lithography shown in Figure S1 (c). Finally, the pentacen layer was deposited in a thermal evaporator, as shown in Figure S1 (d).

X-TEM images and EDS mappings of the HfS_2/pentacene heterojunction

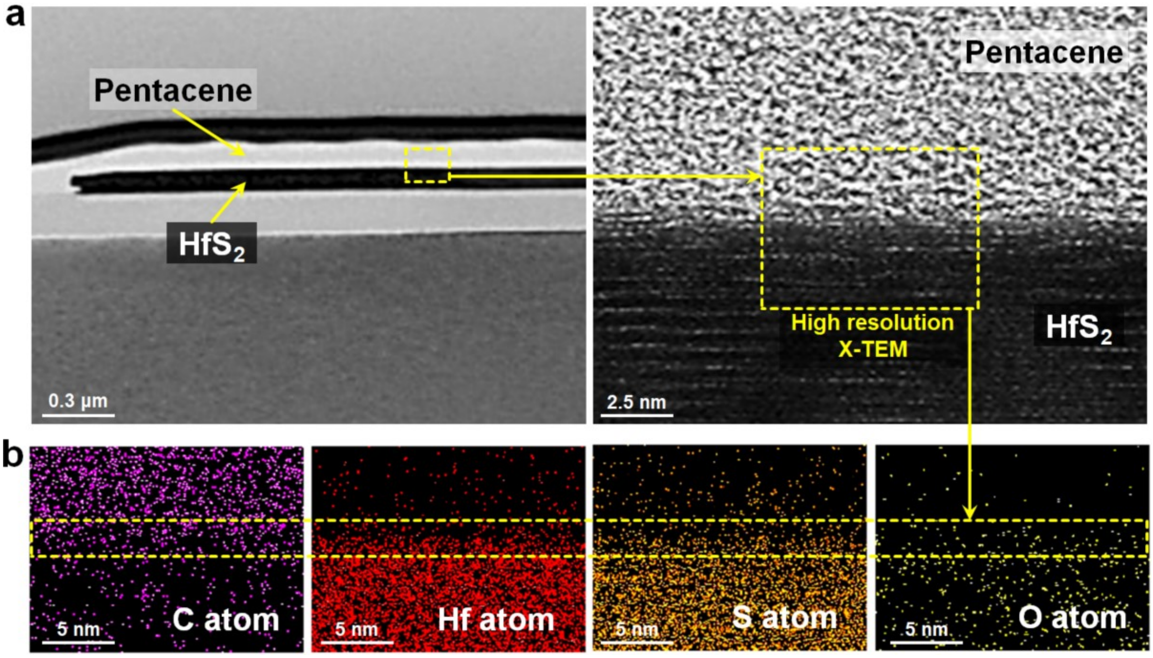

Figure S2. (a) Cross-sectional transmission electron microscopy (X-TEM) images. (b) Energy-dispersive X-ray spectroscopy (EDS) mappings of the HfS_2/pentacene heterojunction structure.

No region suspected of oxidized HfS_2 was found, and the oxygen concentration (O atom) was negligibly low at the HfS_2/pentacene interface.

Energy band structures of HfS_2 and pentacene via UPS measurement

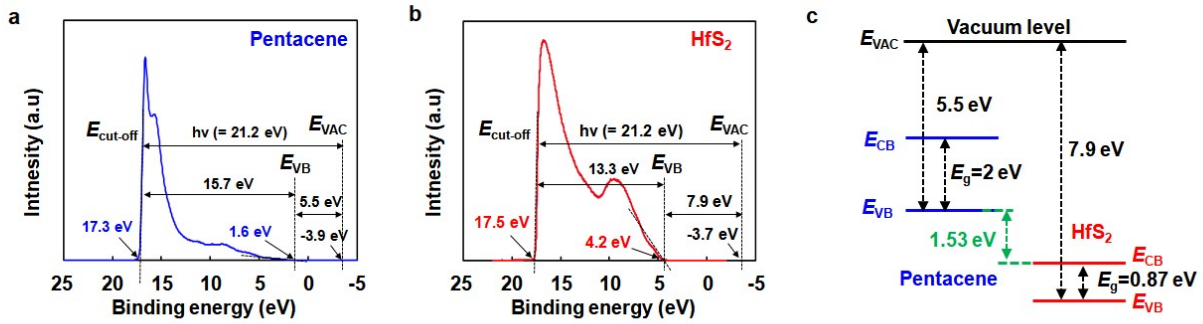

Figure S3. UPS spectrum of (a) pentacene and (b) HfS_2. (c) Energy band alignment of the pentacene and HfS_2 obtained from UPS measurement.

We conducted Ultraviolet photoelectron spectroscopy (UPS) analysis to find the energy band structures for the HfS_2/pentacene heterojunction. However, it is not easy to analyze the binding energies at junction interfaces through the UPS measurements unless the materials forming the junction are very thin. This is because the maximum penetration depth of the beam is only 2.5 nm in used UPS system. Thus, it was impossible to inspect the junction interface region of our heterojunction consisting of 50 nm pentacene and 37 nm HfS_2. The only data we could obtain from the UPS measurements were the energy band properties of the two substances (pentacene and HfS_2), as shown in Figure S3 (a) - (c)[S1]. The data were used to verify once again that the materials have energy band properties suitable for forming a type-III broken heterojunction.

Energy band information and *I-V* characteristic curves of Pentacene–TMDs

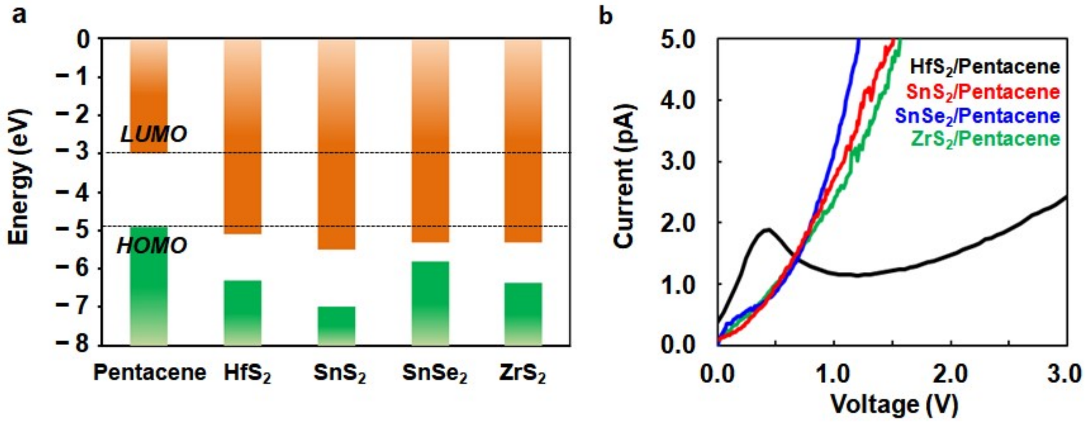

Figure S4. (a) Energy band information of pentacene and vdW TMD materials. (b) Comparison of *I–V* characteristic curves of four kinds of heterojunction diodes.

In Figure S4 (a), the energy band information of organic pentacene and four 2D vdW TMD materials (HfS_2, ZrS_2, SnS_2, and $SnSe_2$) was examined[S2]. Although all the vdW materials were expected to form a broken gap with pentacene, only an HfS_2/pentacene junction experimentally presented the NDR phenomenon, as shown in Figure S4 (b).

Current-voltage characteristics of four different HfS_2/pentacene NDR devices

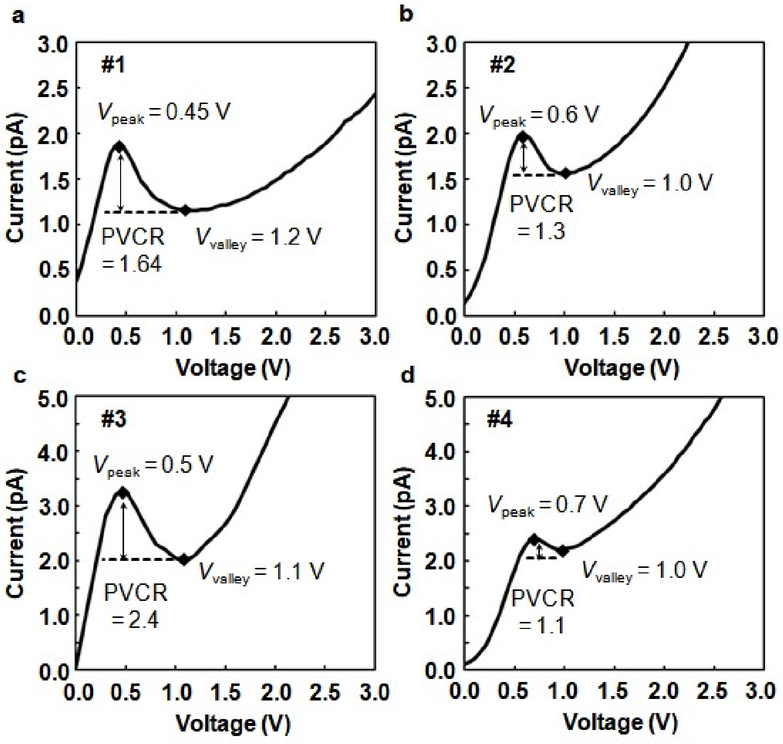

e

Sample Number	V_{PEAK} (V)	V_{VALLEY} (V)	PVCR (A/A)
#1	0.45	1.20	1.64
#2	0.60	1.00	1.30
#3	0.50	1.10	2.40
#4	0.70	1.00	1.10
Average	0.56	1.08	1.63
Standard deviation	0.11	0.10	0.57

Figure S5. (a) - (d) Current-voltage characteristics of HfS_2/pentacene NDR devices. (e) The NDR characteristic parameters extracted from the measured curves of four different NDR device samples.

Figures S5 (a) - (d) present the current-voltage characteristic curves of four different HfS_2/pentacene NDR devices. These four devices were designed to have the same distance of 5 μm between anode and edge of HfS_2/pentacene heterojunction, consequently being expected to cause the same pentacene resistance value of 100 GΩ. The average values of V_{PEAK}, V_{VALLEY}, and PVCR for the four NDR devices were 0.56 V, 1.08 V and 1.63 (A/A), respectively, and the

corresponding standard deviations were 0.11, 0.10 and 0.57, as shown in Figure S5 (e).

Temperature-dependent peak-to-valley current ratio (PVCR)

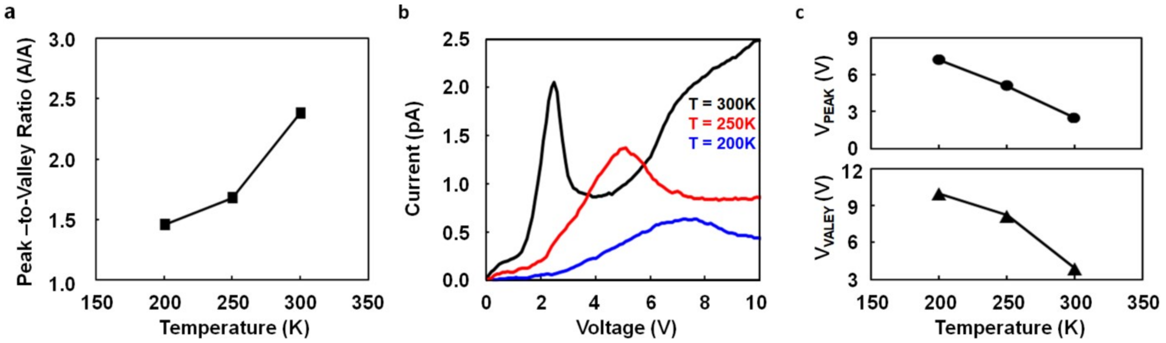

Figure S6. Temperature-dependent electrical characteristics of the HfS_2/pentacene NDR device.

We prepared HfS_2/pentacene NDR device samples and conducted *I-V* measurements twice at three different temperatures of 300, 250, and 200 K. As decreasing the measurement-temperature, the PVCR value was reduced approximately from 2.38 to 1.46 due to an increase in the series resistance of the pentacene region (see Figure S6 (a)). The suppression of carrier generation at a low temperature increased the series resistance, consequently reducing the current level and shifting the peak/valley voltages to the right (see Figures S6 (b) and (c)).

Extraction of sheet resistance values from electrical measurement of HfS_2 and pentacene TFTs

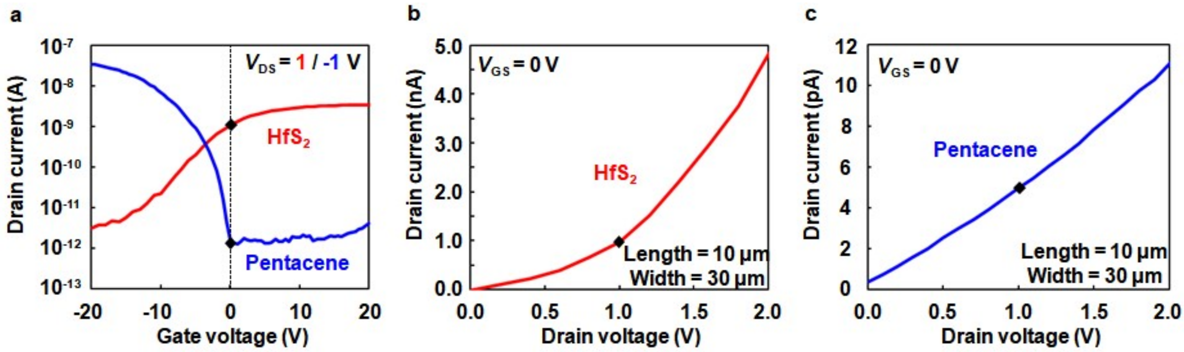

Figure S7. (a) $I_D - V_G$ curves of the HfS_2 and pentacene TFTs at $V_{DS} = 1$ / -1 V. (b) $I_D - V_D$ curve of the HfS_2 TFT at $V_{GS} = 0$ V. (c) $I_D - V_D$ curve of the pentacene TFT at $V_{GS} = 0$ V.

Figure S7 (a) shows the drain current-gate voltage ($I_D - V_G$) characteristics of the HfS_2 and pentacene TFTs at $V_{DS} = 1$ V and -1 V, respectively [S3-S6]. Because the proposed HfS_2/pentacene NDR device operates under zero gate bias condition, the sheet resistance values of HfS_2 and pentacene were extracted from the $I_D - V_D$ curves shown in Figures S7 (b) and (c). From the measured I_D of HfS_2 and pentacene TFTs, which are 0.98 nA and 5 pA, the sheet resistance values of 3 GΩ/sq and 600 GΩ/sq are estimated, respectively. Owing to the larger sheet resistance values of pentacene compared to that of HfS_2, pentacene was selected as a controlling factor for shifting the NDR peak/valley, where we varied the distance between the anode and the edge of HfS_2/pentacene heterojunction.

Carrier-transport mechanism of double-peak NDR device

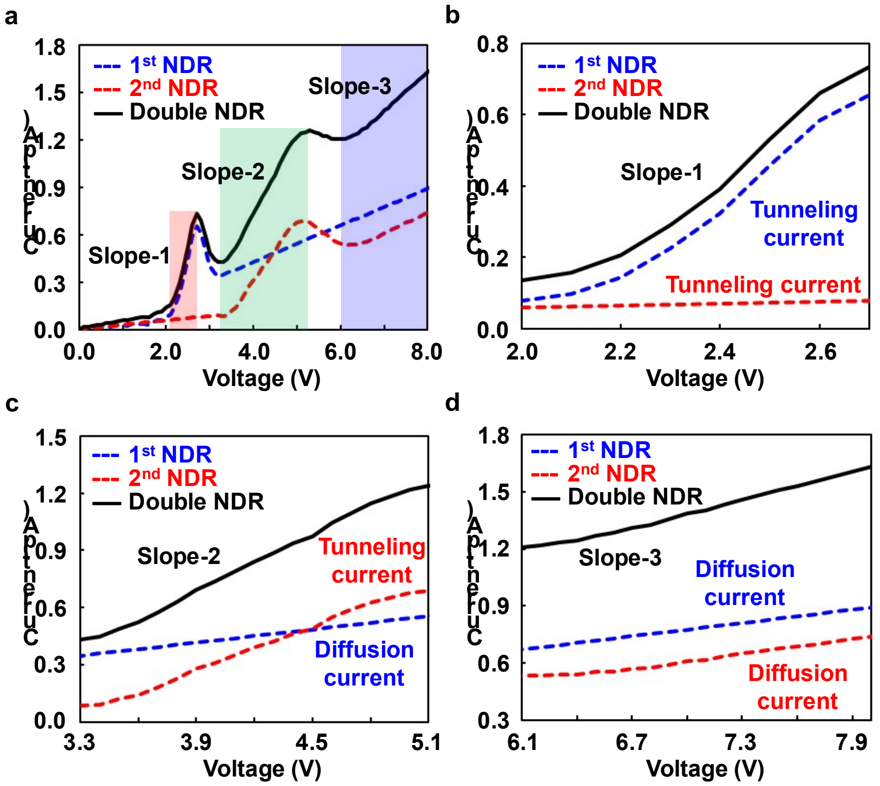

Figure S8. (a) *I*–*V* characteristics of 1st NDR device with R_{lateral} (blue), 2nd NDR device with $R_{\text{lateral}} + R_{\text{vertical}}$ (red), and double-peak NDR device (black). The carrier transport mechanism of the 1st NDR, 2nd NDR, and double-peak NDR devices in (b) slope-1, (c) slope-2 and (d) slope-3 regions, respectively.

Figure S8 (a) presents the *I*–*V* characteristic curve of double-peak NDR device including the predicted characteristic curves of the 1st NDR and 2nd NDR devices. This double-peak NDR curve can be explained by the carrier-transport mechanism based on tunneling and diffusion[S8]. Figure S8 (b) shows the double-peak NDR curve consisting of the tunneling currents of 1st and 2nd NDR paths, resulting in the largest *I*–*V* slope (slope-1 region). The current in the slope-2 region is composed of the diffusion current of 1st NDR path and the tunneling current of 2nd NDR path, as shown in Figure S8 (c), and therefore, the *I*–*V* slope is decreased when compared to the slope-1 region. Lastly, Figure S8 (d) shows the smallest *I*–*V* slope. This is because the current in this slope-3 region is determined by the diffusion currents of 1st and 2nd NDR paths.

Influence of energy bandgap and electron affinity of HfS_2 on the NDR characteristics

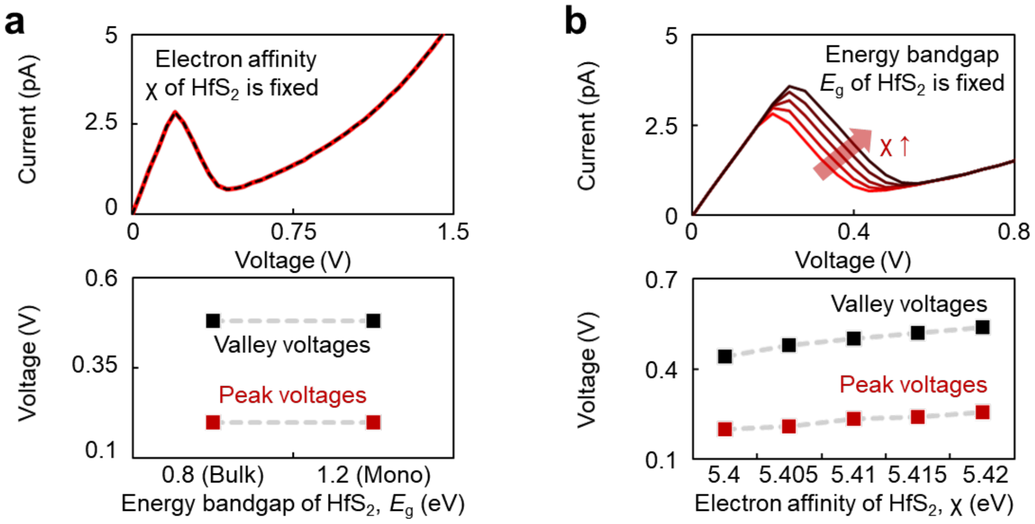

Figure S9. Influence of (a) energy bandgap and (b) electron affinity of HfS_2 on the NDR characteristics.

As depicted in Figure S9, the influence of energy bandgap (E_g) and electron affinity (χ) of HfS_2 on the NDR characteristics was theoretically investigated by using our analytical NDR device model constituting the tunneling and diffusion current equations detailed below.

NDR device's total current (I_{total}) is the sum of two currents: the tunneling (I_{tunnel}) and diffusion (I_{diff}) currents ($I_{total} = I_{tunnel} + I_{diff}$). Here, the tunneling current is based on the Landauer expression for a two-dimensionally confirmed system and is expressed as follows:

$$I_{\mathbf{tunnel}} = \frac{2\pi\alpha q}{h}\int_{E_{C_{\mathbf{HfS2}}}}^{E_{V_{\mathbf{Pentacene}}}} \begin{matrix} DOS_{\mathbf{Pentacene}}(E) \times DOS_{\mathbf{HfS2}}(E) \\ \times [f_{\mathbf{Pentacene}}(E) - f_{\mathbf{HfS2}}(E - qV)]dE \end{matrix}$$

where q is the elementary charge, h is Plank's constant, $E_{V\_Pentacene}$ is the valence band maximum energy of pentacene, α is the fitting parameter, E_{C_HfS2} is the conductance band minimum energy

of HfS_2, and V is the applied voltage. $DOS_{Pentacene}(E)$ and $DOS_{HfS2}(E)$ represent the density of states of pentacene and HfS_2, respectively, and $f_{Pentacene}(E)$ and $f_{HfS2}(E)$ represent the density of states and the Fermi-Dirac distribution functions of pentacene and HfS_2, respectively. The diffusion current is given as

$$I_{diff} = qI_0\left[\exp\left(\frac{V}{nk_BT}\right) - 1\right]$$

where I_0 is the saturation current, n is the ideality factor, k_B is the Boltzmann constant, and T is the temperature. The calculation was performed via MATLAB simulator.

When the χ is fixed and the E_g is varied, no changes in the peak/valley voltages are observed because the broken bandgap is not changed. If the tunneling is considered from the valence band of HfS_2 (filled states) to the valence band of pentacene (empty states), a current level beyond the valley point might be changed. However, we ignore the tunneling between the valence bands of the two materials because it does not affect the peak/valley points. When E_g is fixed and χ is varied, the peak/valley voltages shift to the right because the filled states in the HfS_2 conduction band require a higher voltage to get into pentacene's bandgap region. As χ increases from 5.4 eV to 5.42 eV, the peak/valley voltages increase from 0.2/0.44 V to 0.255/0.54 V. In particular, the peak current level increases because the empty states in the HfS_2 valence band and the filled states in the pentacene conduction band are increased at the equilibrium state.

Specifications of the n-channel transistor in controllable-gain amplifier circuit

a

Geometry of n-channel transistor	Value
Channel length (L)	9 μm
Channel width (W)	10 μm
Oxide thickness (t_{ox})	13 nm

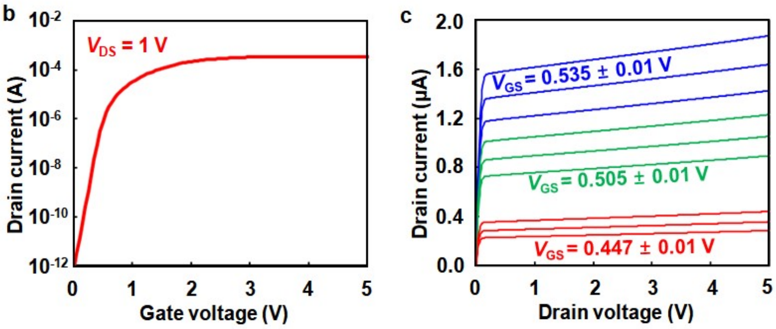

Figure S10. (a) Geometry of the n-channel transistor. (b) $I_D - V_G$ curve of the n-channel transistor at $V_{DS} = 1$ V. (c) $I_D - V_D$ curves in three different ranges of v_{GS} (= $V_{IN} + v_{in}$).

We used the n-channel transistor that is provided by the library of the simulation tool Cadence. The geometric information of the n-channel transistor, comprising channel length, width, and oxide thickness, is listed in Figure S10 (a). Figure S10 (b) shows the $I_D - V_G$ characteristics of the n-channel transistor at $V_{DS} = 1$ V. Figure S10 (c) shows the $I_D - V_D$ curves obtained at three different ranges of v_{GS} (= $V_{IN} + v_{in}$) that were used for the load-line analysis.

Operation of controllable-gain amplifier based on the double-NDR device

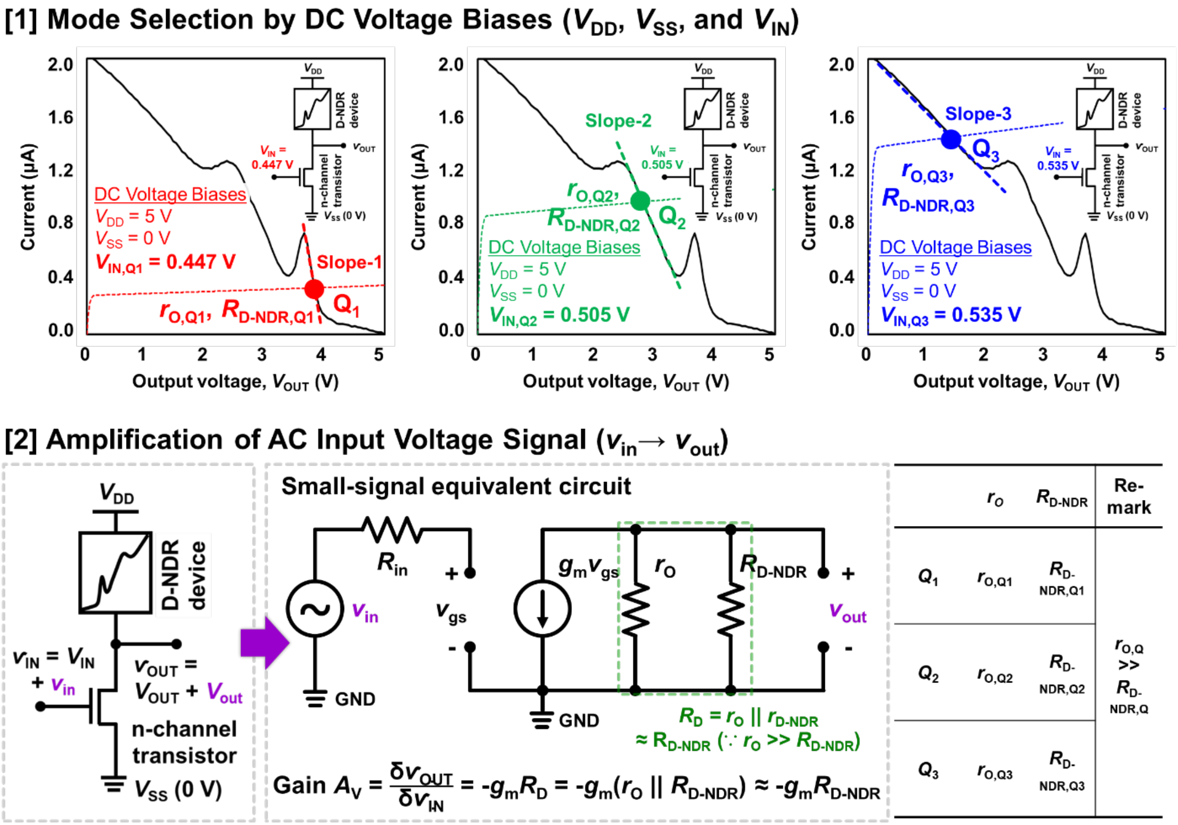

Figure S11. Operation of the controllable-gain amplifier based on the double-NDR device

As depicted in section [1] of Figure S11, the controllable-gain amplifier is stabilized at one of the three operating nodes (Q_1, Q_2, and Q_3) when the DC voltages are applied as follows: {V_{DD}, V_{SS}, $V_{IN,Q}$} = {5 V, 0 V, 0.447 V}, {5 V, 0 V, 0.505 V}, and {5 V, 0 V, 0.535 V} for the nodes Q_1, Q_2, and Q_3, respectively. Here, the output resistance with respect to the channel length modulation of the n-channel transistor and the output resistance of the D-NDR device for the AC input voltage signal analysis are determined as $r_{O,Q1}$ and $R_{D\text{-}NDR,Q1}$ for the node Q_1, $r_{O,Q2}$ and $R_{D\text{-}NDR,Q2}$ for the node Q_2, and $r_{O,Q3}$ and $R_{D\text{-}NDR,Q3}$ for the node Q_3, respectively. Such output resistances are varied by adjusting the DC voltages for the operating mode selection.

Then, to explain the amplification of the AC input voltage signal, as depicted in section [2] of Figure S11, a small-signal equivalent circuit was prepared, where v_{in}, R_{in}, v_{gs}, g_m, R_D, and v_{out}

denote AC input voltage signal, input resistance, gate-source voltage, transconductance of the n-channel transistor, total output resistance, and AC output voltage signal, respectively. Because the output resistance of the n-channel ($r_{O,Q}$) is expected to be larger than that of the D-NDR device ($r_{D\text{-}NDR,Q}$) at every operating node, the total output resistance is approximately $R_{D\text{-}NDR}$ (see Figure S11 inset table). The voltage gain (A_V) of the amplifier is $-g_m R_{D\text{-}NDR}$, which can be controlled according to the mode selection as follows: $\{V_{IN}, v_{in}, A_V\}$ = {0447 V, ± 10 mV, 3.6}, {0.505 V, ± 10 mV, 20.7}, and {0.535 V, ± 10 mV, 37.7} for the nodes Q_1, Q_2, and Q_3, respectively.

Signal amplification at different frequencies

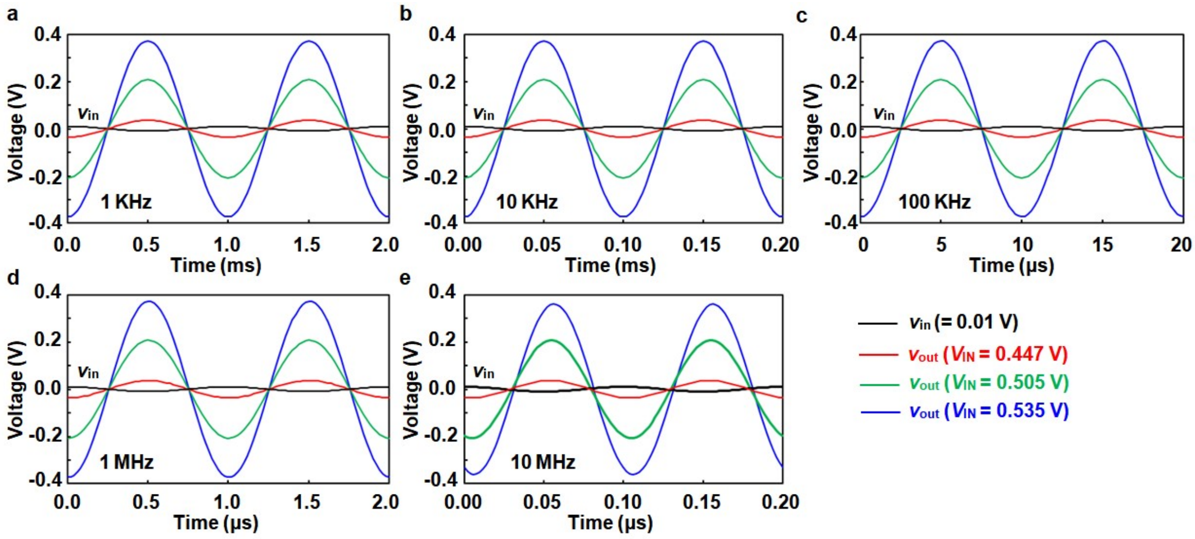

Figure S12. Output AC signals (v_{out}) corresponding to the input AC signal (v_{IN}, black line) for three different DC V_{IN} conditions (0.447/0.505/0.535 V, red/green/blue lines, respectively) at different frequencies: (a) 1 KHz, (b) 10 KHz, (c) 100 KHz, (d) 1 MHz, and (e) 10 MHz.

The proposed controllable-gain amplifier operated well in the 1 kHz – 10 MHz frequency range, as shown in Figure S12.

Double-NDR-device-based ternary inverter

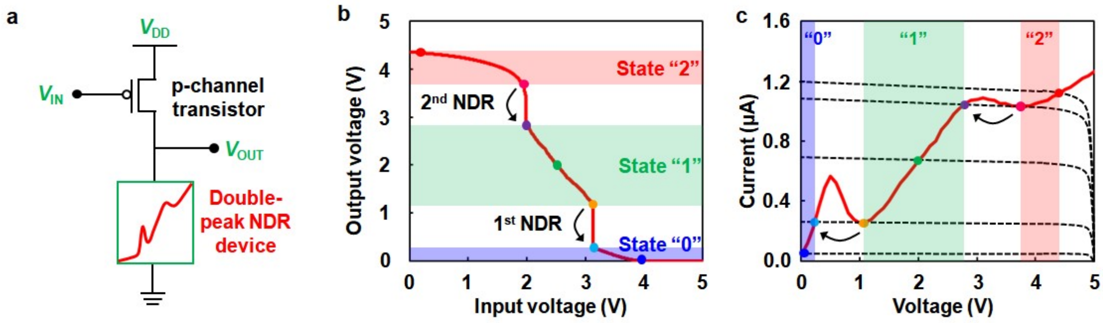

Figure S13. Double-NDR-device-based ternary inverter presenting three logical states. (a) Circuit configuration, (b) V_{IN} vs V_{OUT} characteristic, and (c) load-line analysis of the ternary inverter circuit.

The proposed double-NDR device can be made into a ternary inverter. Figure S13 (a) shows the circuit configuration of the ternary inverter, where the supply (V_{DD}) and input (V_{IN}) voltages were connected to the drain and gate electrodes of the p-channel transistor, respectively. Figure S13 (b) presents the V_{IN} *vs* V_{OUT} characteristic of the ternary inverter. As V_{IN} varies from 0 V to 5 V, V_{OUT} displays three distinct logical states: "2," "1," and "0". The load-line analysis of the ternary inverter circuit under different gate bias conditions is shown in Figure S13 (c). For the state "1," owing to the low slope of the *I*–*V* curve, the distribution of the output voltage is excessively wider than that for the other states.

Performance comparison of various 2D material-based NDR devices

Material	Peak Voltage [V]	PVCR (A/A)	Number of Peaks	Application	Reference
HfS_2/Pentacene	1st V_{PEAK} = 2.7<br>2nd V_{PEAK} = 5.3	1st NDR = 2.2<br>2nd NDR = 1.4	Two	Controllable-gain amplifier	This work
Graphene/*h*-BN/graphene	0.7	1.3	One	Oscillator	[S7]
BP/ReS_2	0.4	4.2	One	Ternary inverter	[S8]
MoS_2/WSe_2	0.5	1.6	One	Ternary inverter	[S9]
BP/$SnSe_2$	0.15 V	1.8	One	-	[S10]
BP/ReS_2 or /HfS_2	1st V_{PEAK} = 0.79<br>2nd V_{PEAK} = 1.03	1st NDR = 1.94<br>2nd NDR = 1.47	Two	Ternary SRAM	[S11]

Table S1. NDR devices benchmarking table

REFERENCES

[S1] W.-H. Kim, W.J. Maeng, M.-K Kim, J. Gatineau, H. Kim. *Journal of The Electrochemical Society*. **2011**, 158, 217-220.

[S2] C. Zhang, C. Gong, Y. Nie, K. Min, C. Liang, Y. J. Oh, H. Zhang, W. Wang, S. L. Hong, L. Colombo, R. M. Wallace, K.J. Cho, *2D Mater*. **2017**, 4, 1, 015026.

[S3] T. Kanazawa, T. Amemiya, A. Ishikawa, V. Upadhyaya, K. Tsuruta, T. Tanaka, Y. Miyamoto, *Sci. Rep*. **2016**, 6, 22277.

[S4] X.-R. Nie, B.-Q. Sun, H. Zhu, M. Zhang, D.-H. Zhao, L. Chen, Q.-Q. Sun, D. W. Zhang, *ACS Appl. Mater. Interfaces.* **2017**, 9, 26996-27003.

[S5] Y.-Y. Lin, D. J. Gundlach, S. F. Nelson, T. N. Jackson, *IEEE Transaction on Electron Device*. **1997**, 44, 8.

[S6] H. L. Cheng, W. Y. Chou, C. W. Kuo, F. C. Tang, T. W. Wang, *Appl. Phys. Lett.* **2006**, 88, 161918.

[S7] A. Mishchenko, J. S. Tu, R. V. Gorbachev, J. R. Wallbank, M. T. Greenaway, V.E. Morozov, S. V. Morozov, M. J. Zhu1, S. L. Wong, F. Withers, C. R. Woods, Y-J. Kim, K. Watanabe, T. Taniguchi, E. E. Vdovin, O. Makarovsky, T. M. Fromhold, V. I. Fal'ko, A.K. Geim, L. Eaves and K. S. Novoselov, *Nat. Nanotech*. **2014**, 9, 808-813.

[S8] J. Shim, S. Oh, D.-H. Kang, S.-H. Jo, M. H. Ali, W.-Y. Choi, K. Heo, J. Jeon, S. Lee, M. Kim, Y. J. Song, J.-H. Park, *Nat. Commun*. **2016**, 7, 13413.

[S9] A. Nourbakhsh, A. Zubair, M. S. Dresselhaus, and Tomás Palacios, *Nano Lett.* **2016**,16, 1359-1366.

[S10] R. Yan, S. Fathipour, Y. Han, B. Song, S. Xiao, M. Li, N. Ma, V. Protasenko, D. A. Muller, D. Jena, H. G. Xing, *Nano. Lett.* **2015**, 15, 5791-5798.

[S11] K.-H. Kim, H.-Y. Park, J. Shim, G. Shin, M. A, J. Koo, G. Yoo, K. Jung, K. Heo, Y. Lee, H.-Y. Yu, K. R. Kim, J. H. Cho, S. Lee and J.-H. Park, *Nanoscale. Horiz*. **2020**, 5, 654-662.
